# Association, effects and validation of polymorphisms within the *NCAPG *- *LCORL *locus located on BTA6 with feed intake, gain, meat and carcass traits in beef cattle

**DOI:** 10.1186/1471-2156-12-103

**Published:** 2011-12-14

**Authors:** Amanda K Lindholm-Perry, Andrea K Sexten, Larry A Kuehn, Timothy PL Smith, D Andy King, Steven D Shackelford, Tommy L Wheeler, Calvin L Ferrell, Thomas G Jenkins, Warren M Snelling, Harvey C Freetly

**Affiliations:** 1 United States Department of Agriculture, Agricultural Research Service, U.S. Meat Animal Research Center, State Spur 18D, Clay Center, NE 68933, USA; 2 Kansas State University, Manhattan, KS, USA; 3 F&J Livestock Consulting, LLC., Saronville, NE, USA

## Abstract

**Background:**

In a previously reported genome-wide association study based on a high-density bovine SNP genotyping array, 8 SNP were nominally associated (*P *≤ 0.003) with average daily gain (ADG) and 3 of these were also associated (*P *≤ 0.002) with average daily feed intake (ADFI) in a population of crossbred beef cattle. The SNP were clustered in a 570 kb region around 38 Mb on the draft sequence of bovine chromosome 6 (BTA6), an interval containing several positional and functional candidate genes including the bovine *LAP3, NCAPG*, and *LCORL *genes. The goal of the present study was to develop and examine additional markers in this region to optimize the ability to distinguish favorable alleles, with potential to identify functional variation.

**Results:**

Animals from the original study were genotyped for 47 SNP within or near the gene boundaries of the three candidate genes. Sixteen markers in the *NCAPG-LCORL *locus displayed significant association with both ADFI and ADG even after stringent correction for multiple testing (P ≤ 005). These markers were evaluated for their effects on meat and carcass traits. The alleles associated with higher ADFI and ADG were also associated with higher hot carcass weight (HCW) and ribeye area (REA), and lower adjusted fat thickness (AFT). A reduced set of markers was genotyped on a separate, crossbred population including genetic contributions from 14 beef cattle breeds. Two of the markers located within the *LCORL *gene locus remained significant for ADG (P ≤ 0.04).

**Conclusions:**

Several markers within the *NCAPG-LCORL *locus were significantly associated with feed intake and body weight gain phenotypes. These markers were also associated with HCW, REA and AFT suggesting that they are involved with lean growth and reduced fat deposition. Additionally, the two markers significant for ADG in the validation population of animals may be more robust for the prediction of ADG and possibly the correlated trait ADFI, across multiple breeds and populations of cattle.

## Background

Feed intake and average daily gain are economically important phenotypes in beef cattle. The ability to genetically select for animals that better suit a producer's needs in terms of the amount of feed an animal consumes and the gain that can be expected would benefit the industry. However, it is critical that markers used to select for feed and growth traits do not adversely affect other traits including meat quality, carcass traits or reproduction.

Quantitative trait loci (QTL) for cattle growth traits such as birth weight, body length, carcass weight, and longissimus muscle area (LMA) have been detected on bovine chromosome 6 in several different populations of animals. Nkrumah et al. [1] discovered a QTL at 42 cM for average daily gain (ADG) in a composite population of steers. Body length at birth and birth weight QTL were identified by Gutiérrez-Gil et al. [2] at 38 and 39 Mb, respectively, in a Charolais × Holstein crossbred population. Several other QTL have been detected for birth weight in this same region [3-6]. In addition to live animal growth, length and weight QTL, others have found that QTL for carcass and meat quality traits are located in this region. Takasuga et al. [7] detected a QTL for carcass weight at 38 cM, Setogutchi et al. [8] found QTL for carcass weight and LMA at 39.1 cM and McClure et al. [6] identified a QTL for ribeye area (REA) at 41 cM. There is clear evidence that the region on BTA6 between 30 and 42 cM is involved in growth and size phenotypes across multiple breeds of cattle.

We have also detected a region on chromosome 6 that is correlated with feed intake and gain located within 37.96 - 38.53 Mb in a crossbred population of cattle [9]. There are seven genes on the Btau 4.0 genome assembly in this region, including leucine aminopeptidase 3 (*LAP3*), mediator complex subunit 28 (*MED28*), *LOC523874*, *C6H4Orf30*, non-SMC condensin I complex, subunit G (*NCAPG*), and *LOC540095 *(bovine ortholog of ligand dependent nuclear receptor corepressor-like, *LCORL*). *LAP3 *is an aminopeptidase that catalyzes the removal of amino acids from intracellular proteins and peptides. *LAP3 *is most highly expressed in the bovine pineal gland, kidney, skin, intestine, mammary, and adipose tissues (UGID: 1959690, UniGene Bt.56962). *MED28 *is a subunit of the Mediator complex that interacts with RNA polymerase II and can interact with co-activators and co-repressors [10]. *MED28 *is most highly expressed in the bovine pineal gland, lyphoreticular tissue, uterus, abomassum, and expressed in lower quantities in the liver, reticulum, intestine and kidney (UGID:1230902, UniGene: Bt.29898). *LOC523874 *is homologous to the human family with sequence similarity 184, member B (*FAM184B*) for which there is little information. Recent SNP association studies suggest that *NCAPG *has a function in cattle growth ([8,11] possibly through a role in cell proliferation [12]. *NCAPG *is expressed in bovine pancreas, skin, intestine, liver, kidney, and ovary (UGID:3093465, UniGene Bt.100379). *LCORL *has recently been associated with human skeletal size [13] and height growth in infancy [14]. *LCORL *is also expressed in the liver, intestine in the bovine fetus and calf, and in the extraembryonic tissue of the bovine fetus (UGID:1484658, UniGene Bt.38533). Based on this information, we chose to evaluate *LAP3*, *NCAPG *and *LCORL *as potential positional candidate genes for cattle feed intake and growth.

The purpose of this study was to further refine the region on chromosome 6 that was previously identified by a SNP association as significant for feed intake and average daily gain in our population of steers [9]. While genetic markers predictive for feed intake and growth may be useful for the identification and selection of animals that are more efficient, attention must be given to the impact of these SNP on other production traits. Accordingly, we evaluated the markers that were highly significant for feed intake and gain for effects on carcass and meat quality traits. Here, we present a number of markers that may be useful for the genetic selection of animals of multiple breeds for feed and gain traits and illustrate that these markers appear to play a role in lean growth and fat deposition.

## Methods

### Animals

All animal procedures were reviewed and approved by the U.S. Meat Animal Research Center (USMARC) Animal Care and Use Committee. Procedures for handling cattle complied with those specified in the Guide for the Care and Use of Agricultural Animals in Agricultural Research and Teaching [15].

### Discovery population

The breeding scheme used for the SNP discovery population of animals with feed efficiency phenotypes is described in Snelling et al. [9]. Briefly, purebred Angus (AN), Hereford (HH), Simmental (SM), Limousin (LM), Charolais (CH), Gelbvieh (GV) and Red Angus (AR) sires were mated by artificial insemination to composite MARC III (1/4 AN, 1/4 HH, 1/4 Pinzgauer, 1/4 Red Poll), AN and HH cows to produce progeny designated as F_1_, born in 1999, 2000, and 2001. Female F_1 _and the 2001-born F_1 _males (AN, HH dams only) were kept for breeding, and mated in multiple-sire pastures to produce 2-, 3-and 4-breed cross progeny designated F_1_^2^. The F_1_^2 ^calves were born in March through May of 2003 to 2007, from 3-year-old and older dams. Male calves were castrated within 24 h after birth. Calves were weaned in September at approximately 165 d of age [9]. Animals were screened and excluded for medical or health issues that may have affected either feed intake or gain phenotypes.

### SNP identification population

Animals (n = 24) used for resequencing of candidate genes *LAP3*, *NCAPG*, and *LCORL *were selected from the discovery population of animals. These animals represented extreme feed intake and gain phenotypes.

### Validation population

Validation animals (n = 406) were derived from progeny in the USMARC continuous Germplasm Evaluation Program [16]. These progeny were part of a breeding program to develop purebred cattle of each of 14 breeds. In addition to the seven mentioned previously, these include Braunvieh, Shorthorn, Brahman, Maine Anjou, Santa Gertrudis, Salers, and Chi-Angus. Sires of each of these 14 breeds were mated to female descendants of cattle in the discovery population. This population did not include full or partial sibs from the discovery population. Approximately 60% of the validation animals were half-bloods sired by these seven new breeds and 40% were half- or three-quarter-bloods from the original seven breeds. Progeny were produced in three breeding seasons (Fall 2007, Spring 2008, and Fall 2008). Animals (n = 29) were removed from the analysis due to chronic diagnoses of pneumonia, foot-rot, and bloat).

### Feed efficiency phenotypes

Discovery population calves were familiarized to the test ration as described in Snelling et al. [9]. Briefly, beginning at 276 ± 15 d of age, individual feed intake measurements were acquired with Calan Broadbent Feeding Systems (American-Calan-Broadbent, Northwood, NH). Animals were provided feed at 0800 h in the morning and were given ad libitum access. The mixed ration on a dry matter basis was 82.0% dry rolled corn, 10.6% ground alfalfa hay, 5.66% Soybean meal, 1.25% limestone, 0.40% urea, 0.06% salt, 0.007% trace minerals, 0.008% Vitamins A, D, and E, and 0.015% Rumensin 80. Feed refusals were obtained each week.

Feed efficiency phenotypes for F_1_^2 ^steers (discovery population) are described in detail in Snelling et al. [9]. Briefly, dry matter intake (DMI) was equal to cumulative dry matter intake for the 140-d feeding period. Average daily feed intake (ADFI) is the average daily dry matter intake (DMI/140). Individual animal quadratic regressions were fitted for body weight on time, and gain was calculated as the difference of BW predicted at 140 d and the intercept [17].

The steers used for the validation study were fed for either 84 or 148 d. Feed intake for those fed for 84 d was measured using an Insentec system (Marknesse, The Netherlands). Diets were similar to those used in the discovery population. Dry matter intake was equal to the total DMI consumed for either the 84- or 148-d period and averaged to a daily basis. A linear regression was used to predict BW in steers fed for 84 d and a quadratic equation was used to predict BW in steers fed for 148 d. Gain for both groups was calculated for the respective feeding periods as described in the discovery population above.

### Meat quality and carcass phenotypes (discovery population)

F_1_^2 ^steers were serially harvested at a commercial processing plant as described by King et al. [18]. USDA yield and quality grade data were obtained by trained USMARC personnel after 36 h in the cooler at 0°C. Wholesale ribs were obtained as described by King et al. [18]. A posterior section of the ribeye was frozen at 14 d postmortem and a 2.54 cm thick steak was cut from the 11^th ^rib region. For slice shear force (SSF), steaks were thawed at 5°C for 24 h then cooked on a conveyorized electric belt grill to a final internal temperature of 71°C as described by Wheeler et al. [19]. SSF was determined as described by Shackelford et al. [20].

### Identification of SNP markers in *LAP3*, *NCAPG* and *LCORL* (SNP identification population)

DNA was isolated from whole blood or buffy coats collected from steers selected from the discovery population of animals. Animals (n = 24) with extreme ADG or ADFI phenotypes were chosen for SNP discovery. Twelve animals with low ADG or ADFI values and twelve animals with high ADG or ADFI values were used. All seven breeds were represented in the animals with high and low phenotypes. None of the breeds were over-represented in the high and low groups. Angus and Hereford breeds each represented ~30% of the breed composition in both groups. Simmental represented 12.5% in the low group and 16% in the high. Composition of the other breeds (Limousin, Gelbvieh, Red Angus, Charolais, MARCIII) ranged from 3-8%.

Primer pairs for amplification of regions of *LAP3, NCAPG *and *LCORL *from genomic DNA were designed using Primer 3 [21] (code available at http://frodo.wi.mit.edu). Cattle sequences used as templates for primer design were obtained from the chromosome 6 Btau 4.0 assembly. Oligonucleotide primers were synthesized by IDT (Integrated DNA Technologies, Coralville, IA). PCR was performed in a DNA engine Dyad^® ^peltier thermal cycler (Bio-Rad, Hercules, CA). PCR reactions included 0.25 U Hot Star *Taq *polymerase (Qiagen, Valencia, CA); 1× supplied buffer; 1.5 mM MgCl_2_; 80 μM dNTPs; 0.33 μM each primer; and 25 ng genomic DNA in 12 μl reactions. Three microliters of the PCR reaction were electrophoresed in 2% agarose gels to determine quality of amplification, and the remainder was prepared for sequencing after treatment with 0.1 U exonuclease I (USB, Cleveland, OH). Sequencing reactions were precipitated with 70% isopropanol and sequenced with an ABI 3730 capillary sequencer (Applied Biosystems, Foster City, CA). Bases were called with Phred and assembled into contigs with Phrap. Polymorphisms were identified using Polyphred and assessed using Consed http://www.phrap.org.

### Genotyping analysis

Multiplex assays for the Sequenom MASSARRAY^® ^instrument were designed with the MASSARRAY^® ^Assay Design 3.0 software (Sequenom, San Diego, CA). Assays were designed for 47 SNPs in and around the *LAP3, NCAPG*, and *LOC540095 *(*LCORL*) gene loci. Each amplification primer contained a 10-base tag to yield amplification products with different masses. Amplicon lengths were between 90 and 120 bp. Reaction conditions were performed as recommended by Sequenom. A total of 1,137 animals with dry matter intake and average daily gain phenotypes and their sires were genotyped and used in the analyses after exclusion.

### Statistical analysis

The data were analyzed using an animal model (MTDFREML) [22] that included the fixed effects of year and barn. Covariates of age and heterosis were also included. Calf and dam breed compositions were modeled with covariates for proportions AN, HH, SM, LI, CH, GV, AR, and MARC III. Covariates for expected calf heterosis were computed from parental breed composition. The number of copies of the minor allele was fitted separately in the association model. Polygenic and breed effects were included to reduce the effects of family structure on breed- and family-specific alleles [23,24]. Variance components for polygenic effects and error were estimated using MTDFREML [22]. Nominal significance values for each SNP were computed. A Bonferroni correction for multiple testing was applied to the *P*-values by multiplying the *P*-value by the number of markers tested (n = 47). Analyses of significant SNP in the validation population followed the same model definition.

### Linkage disequilibrium (LD) analysis

Linkage disequilibrium (r^2^) was defined for the 47 SNP on chromosome 6 using Haploview 4.0 software [25]http://www.broad.mit.edu/mpg/haploview/index.php. Blocks of LD were based on pairwise LD values. Haploview settings were as follows: the exclusion of animals with > 50% missing genotypes, ignoring pairwise comparisons of markers > 500 kb apart, the percentage of genotypes ≥50% and minimum minor allele frequency of 0.001.

## Results

### Candidate gene SNP identification

The genes *LAP3*, *NCAPG *and *LCORL *were chosen as positional and functional candidate genes in this region. The coding regions and some intronic regions of these genes were sequenced and polymorphisms are shown in Table 1. A polymorphism in the *NCAPG *gene identified previously by Setoguchi et al. [8] (p.I442M) was also detected in our population of animals (marker 83714_224 in this study). In addition to this polymorphism, we identified a second non-synonymous alteration in *NCAPG *(p.L877M). A 3 base pair deletion resulting in an in-frame deletion of a glutamic acid (p.E47del) and a non-synonymous alteration (p.D92E) were detected in *LAP3 *(Table 1).

**Table 1:** SNP identified in *LAP3*, *LCORL*, and *NCAPG *genes in crossbred steers.

Gene ^1^	Marker Name^2^	Accession Number	dbSTS _Id	ss #	SNP^3^	Position in Gene^4^	Location on BTA6^5^	For Primer Name	For Primer Seq	Rev Primer Name	Rev Primer Seq
***LAP3***	**79140_312**	**GF102128**	**1233209**	**196003657**	**INDEL**	**p.E47del**	**37964162**	***LAP3-EX2F***	**GCCTTGTTCTTTTGTCCTTGAG**	***LAP3-EX2R***	**TTGGGTTGAAGTAGCCTGATTT**
*LAP3*	79144_139	GF102139	1233220	196003658	S	p.D92E	37966065	*LAP3-EX4F*	ACCAAATTTCTTGGGGAGTTTT	*LAP3-EX4R*	AACACTGCAAGAGGGAACAAAT
*LAP3*	79146_218	GF102161	1233242	196003659	Y	c.504C>T	37967629	*LAP3-EX5F*	GGGAAGAAGTGAAAGCTCAGAA	*LAP3-EX5R*	AAACTGACTAGGGCCATGAGAG
*LAP3*	79150_94	GF102129	1233210	196003660	Y	c.834C>T	37971945	*LAP3-EX7F*	CATTTTTCATTTCCTGCCTTGT	*LAP3-EX7R*	GTAAGCAAAGGGAAGACAGCAT
***LAP3***	**79152_265**	**GF102108**	**1233189**	**196003661**	**Y**		**37977863**	***LAP3-EX8F***	**TGGAAATTTCTTGGTGCTTGTA**	***LAP3-EX8R***	**CTGACTGCTAACCACATTTTGC**
***LAP3***	**79154_194**	**GF102068**	**1233149**	**196003662**	**Y**		**37979109**	***LAP3-EX9F***	**CACACTCCATGTTCCTTCATGT**	***LAP3-EX9R***	**TGCAGGACTTGTTTTCTTCTCA**
***LAP3***	**79154_347**	**GF102068**	**1233149**	**196003663**	**S**		**37979262**	***LAP3-EX9F***	**CACACTCCATGTTCCTTCATGT**	***LAP3-EX9R***	**TGCAGGACTTGTTTTCTTCTCA**
***LAP3***	**79158_103**	**GF102082**	**1233163**	**196003664**	**S**		**37984197**	***LAP3-EX11F***	**AGGATTTGCCAACATCATCTTT**	***LAP3-EX11R***	**CCATGCCTTCTTTTACAAAGCTA**
***LAP3***	**79158_104**	**GF102082**	**1233163**	**196003665**	**R**		**37984198**	***LAP3-EX11F***	**AGGATTTGCCAACATCATCTTT**	***LAP3-EX11R***	**CCATGCCTTCTTTTACAAAGCTA**
***LAP3***	**79160_105**	**GF102096**	**1233177**	**196003666**	**Y**		**37985986**	***LAP3-EX12F***	**ATTTCAGCAAATGCCCTACAAT**	***LAP3-EX12R***	**TTGAAAATGCAAAAACCAGTTG**
***LAP3***	**79162_241**	**GF102069**	**1233150**	**196003667**	**K**	c.*1641T>G	**37986804**	***LAP3-EX13F***	**TGGGGTTAGATGTTGATTTTTG**	***LAP3-EX13R***	**GTGAATATGAGAGCCACACCAG**
***LAP3***	**79162_246**	**GF102069**	**1233150**	**196003668**	**Y**	c.*1646C>T	**37986809**	***LAP3-EX13F***	**TGGGGTTAGATGTTGATTTTTG**	***LAP3-EX13R***	**GTGAATATGAGAGCCACACCAG**
*LAP3*	79162_438	GF102069	1233150	196003669	R	c.*1838A>G	37987001	*LAP3-EX13F*	TGGGGTTAGATGTTGATTTTTG	*LAP3-EX13R*	GTGAATATGAGAGCCACACCAG

***NCAPG***	**83704_234**	**GF110810**	**1241907**	**263198259**	**R**		**38157198**	***NCAPG-EX4-F***	**TCTGTCCTTTTCAAGGGATGTT**	***NCAPG-EX4-R***	**CAGCCACACAATAATGCGATAG**
*NCAPG*	83704_335	GF110810	1241907	263198262	S		38157299	*NCAPG-EX4-F*	TCTGTCCTTTTCAAGGGATGTT	*NCAPG-EX4-R*	CAGCCACACAATAATGCGATAG
*NCAPG*	83704_358	GF110810	1241907	263198260	W		38157322	*NCAPG-EX4-F*	TCTGTCCTTTTCAAGGGATGTT	*NCAPG-EX4-R*	CAGCCACACAATAATGCGATAG
***NCAPG***	**83704_366**	**GF110810**	**1241907**	**263198261**	**R**		**38157330**	***NCAPG-EX4-F***	**TCTGTCCTTTTCAAGGGATGTT**	***NCAPG-EX4-R***	**CAGCCACACAATAATGCGATAG**
***NCAPG***	**83708_361**	**GF110811**	**1241908**	**263198263**	**Y**		**38158932**	***NCAPG-EX6-F***	**GATGGAAGCTAGAGGGGAACTT**	***NCAPG-EX6-R***	**CTTTTCATGGTAGGCCTAATGC**
***NCAPG***	**83712_168**	**GF110812**	**1241909**	**263198264**	**R**	**c.1261A>G**	**38163729**	***NCAPG-EX8-F***	**TTCTCACTTAATGGGGAGCTGT**	***NCAPG-EX8-R***	**AAATTTTAAGCAAAAACCTGCAA**
*NCAPG*	83712_422	GF110812	1241909	263198265	R		38163983	*NCAPG-EX8-F*	TTCTCACTTAATGGGGAGCTGT	*NCAPG-EX8-R*	AAATTTTAAGCAAAAACCTGCAA
*NCAPG*	83714_209	GF110813	1241910	263198266	M	c.1357A>C	38164388	*NCAPG-EX9-F*	TTTCAGAATGTGAATTTTGGCTTA	*NCAPG-EX9-R*	GTGTGAAAGGGACTGGGTTTTA
***NCAPG***	**83714_224**	**GF110813**	**1241910**	**263198267**	**K**	**p.I442M**	**38164403**	***NCAPG-EX9-F***	**TTTCAGAATGTGAATTTTGGCTTA**	***NCAPG-EX9-R***	**GTGTGAAAGGGACTGGGTTTTA**
***NCAPG***	**83724_658**	**GF110818**	**1241915**	**263198273**	**Y**	**c.2158T>C**	**38180790**	***NCAPG-EX14-F***	**ATTTACTTTTGGTGGGGGATGT**	***NCAPG-EX14-R***	**CAACCTCCCAATAGTCATTTCA**
*NCAPG*	83726_315	GF110815	1241912	263198269	R		38192653	*NCAPG-EX15-F*	ATGTAAGGCCTAAAAGGGAGGT	*NCAPG-EX15-R*	GGAAAGAGCCAAATAATTCAGG
***NCAPG***	**83730_778**	**GF110816**	**1241913**	**263198270**	**M**	**p.L877M**	**38195339**	***NCAPG-EX17-F***	**GAGCGTGAATGGTTCAGTACAC**	***NCAPG-EX17-R***	**ACTTCAAGAAATGAGCACCAAA**
***NCAPG***	**83732_316**	**GF110819**	**1241916**	**263198271**	**R**		**38195743**	***NCAPG-EX18-F***	**AGTATTTGGTGCTCATTTCTTGA**	***NCAPG-EX18-R***	**TCCCAAGTGAATTTAATTAGAAAAA**

*LCORL*	79205_443	GF102113	1233194	196003679	R		38233294	*LOC540095-EX6F*	CCTATGTAGTGCCTTCCCAGTC	*LOC540095-EX6R*	CTCGTCCTGCTTCTTAGTTTGT
***LCORL***	**81441_243**	**GF102084**	**1233165**	**196003694**	**Y**		**38255270**	***LOC540095-IN4.4F***	**GCATGAATGACAAAACTGTGCT**	***LOC540095-IN4.4R***	**CATTTTGCCCTTAAGCCTTCTA**
*LCORL*	81439_210	GF102101	1233182	196003693	R		38257174	*LOC540095-IN4.3F*	TTTGCCTTCAGTTCTCTTAGGC	*LOC540095-IN4.3R*	TTGCAAAATTATGGCATTTCAC
***LCORL***	**81435_188**	**GF102143**	**1233224**	**196003692**	**Y**		**38284737**	***LOC540095-IN4.1F***	**TAGCCTGACTGCATCCATCTAA**	***LOC540095-IN4.1R***	**GGAAATCCCTGGTTAAGAATCC**
*LCORL*	79197_655	GF110814	1241911	263198268	R		38327100	*LOC540095-EX2F*	TCTCACGTAGAGTGTATGGATAAGC	*LOC540095-EX2R*	GAGTTCCAGGCTGCCTATATCA
*LCORL*	81433_176	GF110817	1241914	263198272	Y		38314844	*LOC540095-IN3.1F*	GCAGGTGAAAATCCCAATACAC	*LOC540095-IN3.1R*	GGGCCAAACTAGCCTTATTTCT
***LCORL***	**81419_461**	**GF102100**	**1233181**	**196003691**	**M**		**38342145**	***LOC540095-IN1.9F***	**GACTTCAAATTTTTGCCCAGAG**	***LOC540095-IN1.9R***	**GGTGTTCTTACCCTGTCTCAGC**
*LCORL*	81413_159	GF102115	1233196	196003686	S		38359337	*LOC540095-IN1.6F*	AGGATCAACCATTAGGATGTGC	*LOC540095-IN1.6R*	AACTGGGAAGAGAGCAAGTGAG
*LCORL*	81413_221	GF102115	1233196	196003684	W		38359399	*LOC540095-IN1.6F*	AGGATCAACCATTAGGATGTGC	*LOC540095-IN1.6R*	AACTGGGAAGAGAGCAAGTGAG
*LCORL*	81413_226	GF102115	1233196	196003687	W		38359404	*LOC540095-IN1.6F*	AGGATCAACCATTAGGATGTGC	*LOC540095-IN1.6R*	AACTGGGAAGAGAGCAAGTGAG
*LCORL*	81413_231	GF102115	1233196	196003689	M		38359409	*LOC540095-IN1.6F*	AGGATCAACCATTAGGATGTGC	*LOC540095-IN1.6R*	AACTGGGAAGAGAGCAAGTGAG
*LCORL*	81413_238	GF102115	1233196	196003690	W		38359416	*LOC540095-IN1.6F*	AGGATCAACCATTAGGATGTGC	*LOC540095-IN1.6R*	AACTGGGAAGAGAGCAAGTGAG
***LCORL***	**81405_282**	**GF102099**	**1233180**	**196003685**	**M**		**38376731**	***LOC540095-IN1.2F***	**TCGGGTCCTCTTTTACTGTCAT**	***LOC540095-IN1.2R***	**CTTACCACGATCTCCTTTCCAC**

^1 ^SNP in bold were genotyped using the Sequenom MassArray System.

^2 ^Marker name in USMARC database.

^3 ^IUB Codes for SNP are K = G/T, M = A/C, R = A/G, S = C/G, W = A/T, Y = C/T.

^4 ^Position based on NM_174098 Bos taurus *leucine aminopeptidase 3 *(*LAP3*) mRNA; NM_001102376 Bos taurus *non-SMC condensin I complex, subunit G *(*NCAPG*), mRNA; and NM_001192357 Bos taurus *ligand dependent nuclear receptor corepressor-like *(*LCORL*) mRNA.

^5 ^Position based on the Btau 4.0 genome assembly.

### Discovery population analyses

A total of 21 out of the 39 SNP identified within the *LAP3 *(n = 9), *NCAPG *(n = 8) and *LCORL *(n = 4) genes were multiplexed into Sequenom genotyping assays. In addition to these, another 26 publicly available SNP located up- and down-stream of these genes that were not used on the BovineSNP50 BeadChip [26-28], were also placed into the genotyping assays. The population of steers genotyped previously [9] with the BovineSNP50 BeadChip (n = 1,066) and their sires (n = 71) were genotyped with these markers (Table 2).

**Table 2:** SNP marker associations and estimated effects for ADFI and ADG in the discovery population of crossbred steers.

						Average Daily Feed Intake (ADFI)			Average Daily Gain (ADG)		
						
Marker^1^	Gene^2^	Position^3^	IUB	# Genotypes^4^	MAF	Effect^5^	*P*-value	Correction^6^	Effect	*P*-value	Correction
ARS-BFGL-NGS-77584		37855236	R	1182	G = 0.10	-0.026	0.8	NS	-0.0078	0.7	NS
After2Run10KSet7323		37856744	Y	1075	T = 0.30	-0.14	0.01	NS	-0.029	0.01	NS
Hapmap31601-BTC-034240		37859827	R	1076	G = 0.28	-0.15	0.005	NS	-0.033	0.004	NS
Hapmap28705-BTC-034230		37862818	S	1090	G = 0.31	-0.14	0.010	NS	-0.033	0.003	NS
Hapmap25169-BTC-034210		37870369	R	1186	A = 0.30	-0.15	0.006	NS	-0.033	0.003	NS
Hapmap30444-BTC-052947		37875093	Y	1164	C = 0.49	0.16	0.004	NS	0.036	0.002	NS
After2Run10KSet5598		37875791	Y	1186	C = 0.30	-0.14	0.01	NS	-0.032	0.004	NS
Hapmap32743-BTC-052998		37879284	R	1011	A = 0.23	-0.073	0.2	NS	-0.024	0.04	NS
Hapmap23977-BTC-053017		37880952	M	1176	A = 0.30	-0.15	0.006	NS	-0.032	0.005	NS
79140_312	*LAP3*	37964162	INDEL	1165	DEL = 0.09	0.11	0.2	NS	-0.013	0.5	NS
Hapmap56977-ss46527055	*LAP3*	37967630	R	1192	A = 0.13	-0.051	0.5	NS	-0.013	0.4	NS
79152_265	*LAP3*	37977863	Y	1181	C = 0.33	-0.020	0.7	NS	-0.021	0.05	NS
79154_194	*LAP3*	37979109	Y	1078	T = 0.38	-0.0072	0.9	NS	-0.021	0.06	NS
79154_347	*LAP3*	37979262	S	1098	C = 0.34	-0.022	0.7	NS	-0.021	0.04	NS
79158_103	*LAP3*	37984197	S	1193	G = 0.34	0.026	0.6	NS	0.021	0.05	NS
79158_104	*LAP3*	37984198	R	1190	G = 0.33	-0.021	0.7	NS	-0.020	0.07	NS
79160_105	*LAP3*	37985986	Y	1127	T = 0.34	-0.0086	0.9	NS	-0.019	0.08	NS
79162_241	*LAP3*	37986804	K	1124	T = 0.37	-0.0064	0.9	NS	-0.22	0.05	NS
79162_246	*LAP3*	37986809	Y	1197	C = 0.34	-0.020	0.7	NS	-0.21	0.04	NS
ARS-BFGL-NGS-44699		38011603	S	1183	C = 0.10	-0.085	0.3	NS	-0.021	0.2	NS
BTA-100891		38076964	Y	1063	T = 0.26	-0.083	0.1	NS	-0.028	0.008	NS
83704_234^7^	*NCAPG*	38157198	R	1085	A = 0.42	0.18	0.0006	0.03	0.046	2.E-05	0.0009
83704_366	*NCAPG*	38157330	R	1187	G = 0.12	-0.11	0.2	NS	-0.013	0.4	NS
83708_361	*NCAPG*	38158932	Y	1092	C = 0.41	0.17	0.001	0.05	0.045	4.E-05	0.002
83712_168^7^	*NCAPG*	38163729	R	1004	G = 0.47	-0.16	0.003	NS	-0.045	9.E-05	0.004
83714_224^7^	*NCAPG*	38164403	K	1132	G = 0.34	0.21	8.E-05	0.004	0.059	1.E-07	5.E-06
83724_658	*NCAPG*	38180790	Y	1100	C = 0.41	0.17	0.001	0.05	0.045	5.E-05	0.002
83730_778	*NCAPG*	38195339	M	1022	A = 0.42	0.17	0.003	NS	0.058	1.E-06	0.00005
83732_316	*NCAPG*	38195743	R	1050	G = 0.42	0.14	0.008	NS	0.044	1.E-04	0.005
Hapmap24122-BTC-041153		38217816	K	1193	G = 0.35	0.22	1.E-05	0.0005	0.059	2.E-08	9.E-07
Hapmap24041-BTC-041151		38220879	K	1192	T = 0.35	0.22	1.E-05	0.0005	0.059	2.E-08	9.E-07
Hapmap31609-BTC-041148		38223329	R	1192	A = 0.38	0.17	0.0005	0.02	0.045	1.E-05	0.0005
Hapmap28154-BTC-041134^8^	*LCORL*	38228679	R	935	C = 0.38	0.18	0.0003	0.01	0.045	2.E-05	0.0009
Hapmap27082-BTC-041131	*LCORL*	38233962	R	1121	G = 0.37	0.18	0.0002	0.009	0.047	4.E-06	0.0002
Hapmap26855-BTC-041123	*LCORL*	38239191	R	1075	G = 0.33	0.21	8.E-05	0.004	0.058	1.E-07	5.E-06
Hapmap23341-BTC-041113	*LCORL*	38250098	K	1161	T = 0.36	0.21	4.E-05	0.002	0.056	1.E-07	5.E-06
81441_243	*LCORL*	38255270	S	822	G = 0.28	-0.24	5.E-06	0.0002	-0.058	2.E-07	9E-06
81435_188	*LCORL*	38284737	Y	892	C = 0.38	0.17	0.0004	0.02	0.042	3.E-05	0.001
**Hapmap28589-BTC-041045^9^**	*LCORL*	**38300298**	**S**	**1190**	**G = 0.33**	**0.22**	**2.E-05**	**0.0009**	**0.061**	**1.E-08**	**5.E-07**
Hapmap33629-BTC-041038	*LCORL*	38313142	Y	999	T = 0.30	0.22	2.E-05	0.0009	0.059	5.E-08	2.E-06
Hapmap28835-BTC-041022	*LCORL*	38326993	S	1061	G = 0.18	-0.12	0.07	NS	-0.027	0.04	NS
81419_461	*LCORL*	38342145	M	1058	C = 0.05	-0.17	0.1	NS	-0.046	0.04	NS
Hapmap33694-BTC-041005	*LCORL*	38342183	R	1190	G = 0.16	-0.10	0.1	NS	-0.038	0.005	NS
**81405_282^9^**	***LCORL***	**38376731**	**M**	**1196**	**A = 0.33**	**0.22**	**2.E-05**	**0.0009**	**0.061**	**1.E-08**	**5.E-07**
Hapmap33650-BTC-073421		38409471	Y	1041	C = 0.40	-0.14	0.004	NS	-0.036	0.0003	0.01
Hapmap31943-BTC-050642		38427989	Y	1181	C = 0.08	-0.11	0.2	NS	-0.039	0.02	NS
BTB-00249053		38481012	Y	1183	T = 0.24	-0.12	0.03	NS	-0.029	0.01	NS

^1 ^Name of publicly available markers from the following references: Van Tassell CP et al. [26], Bovine HapMap Consortium et al. [27], and Bovine Genome Sequencing and Analysis Consortium et al. [28]

^2 ^SNP located within a gene locus are presented in this column. The gene loci for LAP3, NCAPG, and LCORL are: 37,961,725 - 37,987,164 bp, 38,153,047-38,199,153 bp, and 38,227,955-38,378,385 bp on the Btau 4.0 genome assembly.

^3 ^Position based on the Btau 4.0 Bovine Genome Assembly.

^4 ^Number of genotypic calls used for statistical analysis.

^5 ^ADFI and ADG effects are displayed as kg/d. Effects are presented for the minor allele.

^6 ^Correction for multiple testing performed by multiplying the *P*-values by the number of markers tested (n = 47). NS = not significant, *P*-value > 0.05.

^7 ^Markers evaluated in Setoguchi et al. [8] and Eberlein et al. [11].

^8 ^Marker evaluated in Charolais and German Holstein population in Eberlein et al. [11].

^9 ^Markers in bold font, Hapmap28589-BTC-041045 and 81405_282, were significantly associated with ADG in the validation population of animals (*P *= 0.04 and 0.03, respectively).

Marker associations for ADFI and ADG are presented in Table 2. A Bonferroni correction for multiple testing was applied to the *P*-values obtained from the genotyping analysis (Table 2). *P*-values were multiplied by the number of markers tested (n = 47) to obtain a corrected *P*. After correction, 16 markers between 38.16 and 38.37 Mb were significantly associated with ADFI (*P *< 0.05) and 20 markers between 38.16 and 38.41 Mb were associated with ADG (*P *< 0.05).

Two markers within the *NCAPG *gene locus were associated (after multiple correction) with ADFI (*P *≤ 0.02) and seven were associated with ADG (*P *= 0.003; Table 2); one (83714_224) was the same marker previously identified by Setoguchi et al. [8] (p.I442M). However, several other markers were identified with lower *P*-values for ADFI and ADG (Table 2).

To determine whether these markers may adversely affect carcass and meat traits, they were analyzed for association with hot carcass weight (HCW), adjusted fat thickness (AFT), marbling score (MARB), ribeye area (REA), and SSF. The phenotypic data is presented in Table 3 and includes: number of animals, mean with standard deviation, and range of phenotypes. The effects and association between meat quality and carcass traits for the markers on BTA6 are presented in Table 4. The alleles for positive effects or increases in ADFI and ADG were associated with positive effects or higher HCW and REA. These same alleles had negative effects or lower AFT. Marbling and SSF were not significant for any of the markers tested (Table 4).

**Table 3:** Description of the USMARC resource population carcass and meat quality phenotypic means.

Phenotype^1^	N	Mean^2^	Range
Hot Carcass Weight (kg)	1066	353 ± 34.6	246.8 - 468.6
Adjusted Fat Thickness (cm)	1066	1.27 ± 0.5	0.1 - 3.3
Marbling (MSU)^3^	1066	531.4 ± 91.8	300 - 910
Ribeye Area (cm^2^)	1066	78.7 ± 8.4	56.1 - 116.8
Slice Shear Force (kg)	1066	12.4 ± 3	6.48 - 27.38

^1 ^Slice shear force was measured at 14 d postmortem.

^2 ^Mean and standard deviation for each phenotype.

^3 ^MSU = Marbling score unit. 400 = Slight^00^, 500 = Small^00^, 600 = Modest^00^.

**Table 4:** Association and effects of SNP significant for ADG on BTA6 with meat quality and carcass traits in the discovery population of crossbred steers.

		Adjusted Fat Thickness(cm)			Hot Carcass Weight (kg)			Ribeye Area (cm^2^)			Marbling (MSU)^1^			Slice Shear Force		
		
Marker Name^2^	Minor Allele	Effect^3^	SE	*P*	Effect^3^	SE	*P*	Effect^3^	SE	*P*	Effect^3^	SE	*P*	Effect^3^	SE	*P*
83704_234^4^	A	-0.102	0.0245	3.8E-05	8.69	1.634	1.8E-07	1.99	0.3714	1.4E-07	-2.45	4.073	0.55	-0.060	0.149	0.69
83708_361	C	-0.095	0.0247	0.0001	7.84	1.648	2.7E-06	1.83	0.3748	1.4E-06	-2.86	4.110	0.49	-0.081	0.151	0.59
83712_168^4^	G	-0.086	0.0263	0.001	8.05	1.753	5.9E-06	1.83	0.3986	6.0E-06	-5.33	4.371	0.22	-0.012	0.160	0.94
83714_224^4^	G	-0.125	0.0250	9.2E-07	10.39	1.671	1.3E-09	2.33	0.3798	1.9E-09	-6.94	4.165	0.10	-0.031	0.153	0.84
83724_658	C	-0.096	0.0250	0.0002	8.25	1.671	1.2E-06	1.98	0.3801	3.1E-07	-4.23	4.168	0.31	-0.073	0.153	0.63
83730_778	A	-0.100	0.0271	0.0002	9.15	1.807	6.2E-07	2.20	0.4110	1.5E-07	-4.47	4.506	0.32	-0.086	0.166	0.60
83732_316	G	-0.097	0.0256	0.0002	8.10	1.711	3.0E-06	1.92	0.3890	1.2E-06	-3.27	4.266	0.44	-0.084	0.157	0.59
Hapmap24122-BTC-041153	G	-0.120	0.0236	5.3E-07	10.56	1.577	7.4E-11	2.43	0.3584	4.3E-11	-4.47	3.931	0.26	-0.075	0.144	0.60
Hapmap24041-BTC-041151	T	-0.119	0.0235	6.3E-07	10.38	1.570	1.2E-10	2.39	0.3569	6.9E-11	-3.83	3.914	0.33	-0.085	0.143	0.55
Hapmap31609-BTC-041148	A	-0.108	0.0232	4.7E-06	8.56	1.552	6.3E-08	2.02	0.3527	2.0E-08	-3.45	3.868	0.37	-0.10	0.142	0.48
Hapmap28154-BTC-041134^5^	C	-0.108	0.0235	5.4E-06	8.31	1.568	1.9E-07	1.87	0.3563	2.3E-07	-2.64	3.908	0.50	-0.084	0.143	0.56
Hapmap27082-BTC-041131	G	-0.108	0.0232	4.5E-06	8.59	1.553	5.6E-08	1.98	0.3527	3.6E-08	-3.93	3.869	0.31	-0.091	0.142	0.52
Hapmap26855-BTC-041123	G	-0.127	0.0244	2.8E-07	10.41	1.631	4.9E-10	2.12	0.3705	1.9E-08	-6.65	4.064	0.10	-0.056	0.149	0.71
Hapmap23341-BTC-041113	T	-0.114	0.0236	1.8E-06	10.16	1.575	3.2E-10	2.26	0.3579	6.7E-10	-4.34	3.925	0.27	-0.077	0.144	0.59
81441_243	G	0.112	0.0248	7.9E-06	-10.59	1.658	4.7E-10	-2.25	0.3766	5.2E-09	1.59	4.131	0.70	0.078	0.151	0.61
81435_188	C	-0.099	0.0231	2.2E-05	8.23	1.545	1.7E-07	1.71	0.3511	1.6E-06	-4.85	3.851	0.21	-0.087	0.141	0.54
Hapmap28589-BTC-041045	G	-0.130	0.0240	9.4E-08	10.63	1.601	1.0E-10	2.33	0.3640	4.3E-10	-5.86	3.992	0.14	-0.044	0.146	0.77
Hapmap33629-BTC-041038	T	-0.127	0.0245	3.6E-07	10.30	1.639	8.4E-10	2.10	0.3721	3.4E-08	-6.45	4.082	0.11	-0.065	0.149	0.66
81405_282	A	-0.130	0.0242	1.4E-07	10.84	1.619	7.2E-11	2.38	0.3679	3.1E-10	-5.61	4.035	0.17	-0.030	0.148	0.84
Hapmap33650-BTC-073421	C	0.054	0.0232	0.02	-7.26	1.546	3.7E-06	-1.35	0.3514	0.0001	5.01	3.854	0.19	0.043	0.141	0.76

^1 ^MSU = Marbling score unit. 400 = Slight^00^, 500 = Small^00^, 600 = Modest^00^.

^2 ^Name of publicly available markers from the following references: Van Tassell CP et al. [26], Bovine HapMap Consortium et al. [27], and Bovine Genome Sequencing and Analysis Consortium et al. [28].

^3 ^Effects of phenotypes are displayed in the following units: AFT = cm, HCW = kg, REA = cm^2^, Marbling = MSU, and SSF = kg. The effects presented correspond to the minor allele.

^4 ^Markers evaluated in Setoguchi et al. [8] and Eberlein [11].

^5 ^Marker evaluated in Charolais and German Holstein population in Eberlein et al. [11].

### Validation population analysis

**Figure 1:**
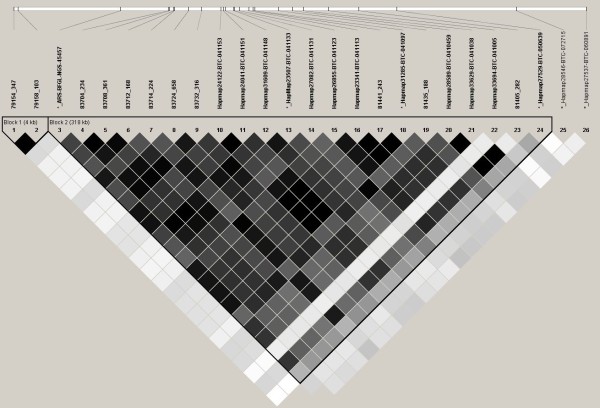
**Haploview display of LD of the 26 genetic markers on chromosome 6 that were genotyped on the validation population of crossbred steers (n = 406)**. Pairwise LD in r^2 ^is shown between markers with shading. The darker shading represents higher LD, black diamonds are r^2 ^= 1. Markers in bold signify those that are located in a haplotype block. Markers with *_ prior to the marker name are markers from the Illumina BovineSNP50 BeadChip that were associated with ADG in the discovery population of animals [9].

**Figure 2:**
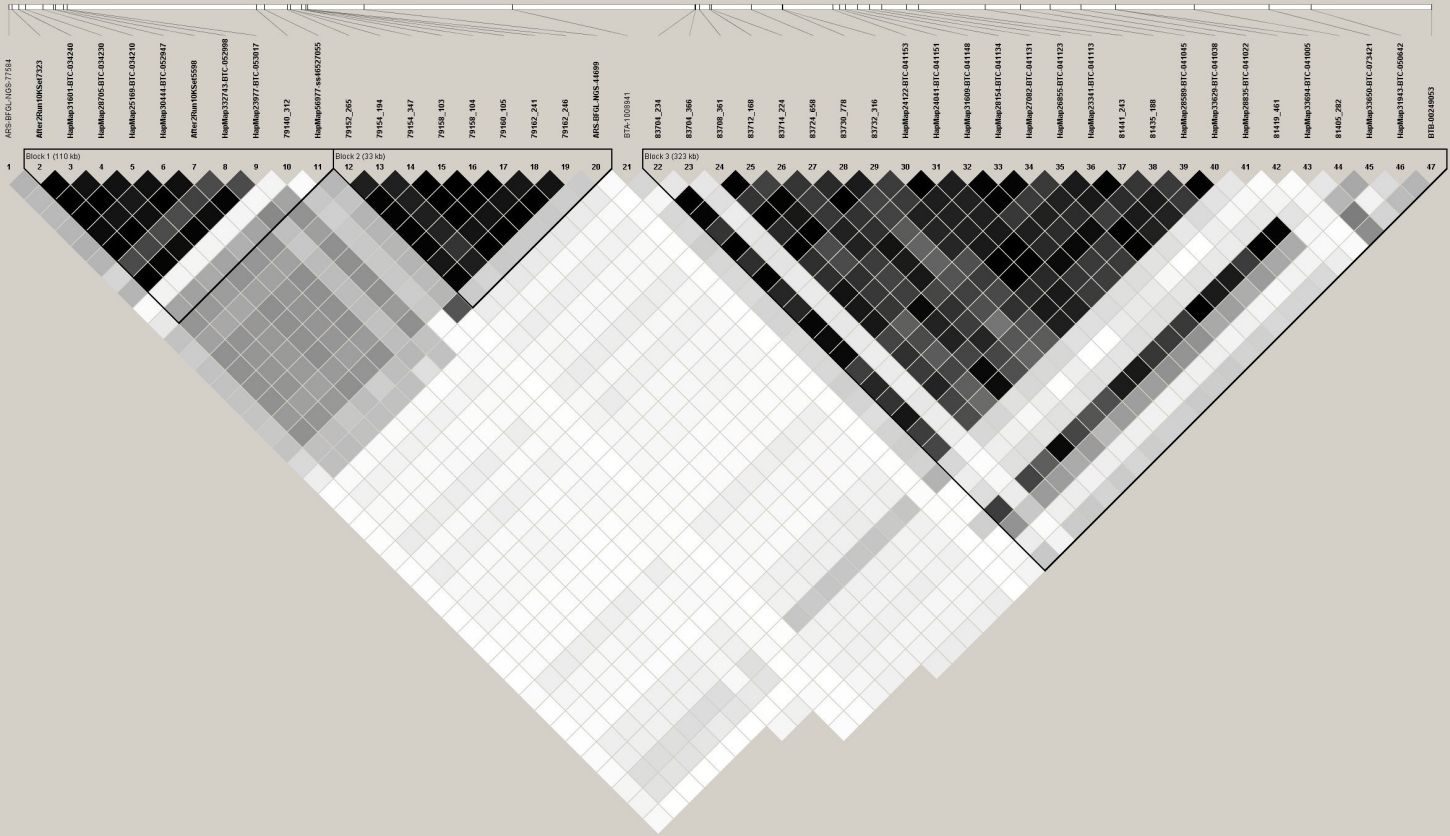
**Haploview display of LD of the genetic markers on chromosome 6 genotyped on the SNP discovery population of crossbred steers (n = 1,066)**. Pairwise LD in r^2 ^is shown between markers by shading. The darker shading represents higher LD, black diamonds are r^2 ^= 1. Markers in bold signify those that are located in a haplotype block.

Twenty of the markers genotyped in the discovery population were genotyped in a validation population of crossbred animals (n = 406; Table 5). This set of markers included two markers in the *LAP3 *gene locus, 6 in *NCAPG *and 9 in the *LCORL *gene loci. Of the 20 markers, 13 and 17 were associated with ADFI and ADG, respectively, after Bonferroni correction in the discovery population. In addition to these 20 markers, six markers from the Illumina BovineSNP50 BeadChip that were nominally significant ADG (4 were also nominally significant for ADFI) in the discovery population were also genotyped in the validation population of animals. Association analysis produced two markers (Hapmap28589-BTC-041045, *P *= 0.04 and 81405_282, *P *= 0.03) that were associated with ADG in these animals (Table 5). Both markers are located within the *LCORL *gene locus. Marker 83714_224 (or p.I422M) [8], in *NCAPG *was not significant in this population (*P *= 0.09). Genotypes were analyzed with Haploview for linkage disequilibrium (LD) in this population of animals (Figure 1). The LD between marker 83714_224 in *NCAPG *and 81405_282 in LCORL was lower at r^2 ^= 0.89 than the LD between these two markers in the SNP discovery population (r^2 ^= 0.96) (Figure 2).

**Table 5:** SNP marker associations and estimated effects for ADG in the validation population of crossbred steers (n = 406).

						Average Daily Gain (ADG)		
						
Marker^1^	Illumina BeadChip^2^	Rs#	Position^3^	IUB	MAF	Effect^5^	*SE*	*P*-value
79154_347			37979262	S	C = 0.34	-0.016	0.0183	0.4
79158_103			37984197	S	G = 0.35	0.019	0.0183	0.3
ARS-BGFL-NGS-45457	✓	rs109447543	38102327	W	A = 0.42	0.029	0.0178	0.1
83704_234			38157198	R	A = 0.49	0.018	0.0166	0.3
83708_361			38158932	Y	C = 0.49	-0.0015	0.0194	0.9
83712_168			38163729	R	A = 0.47	-0.0029	0.0186	0.9
83714_224			38164403	K	G = 0.42	0.031	0.0179	0.09
83724_658			38180790	Y	T = 0.49	-0.023	0.0171	0.2
83732_316			38195743	R	G = 0.49	0.0042	0.0205	0.8
Hapmap24122-BTC-041153			38217816	K	G = 0.43	0.021	0.0175	0.2
Hapmap24041-BTC-041151			38220879	K	T = 0.44	0.022	0.0175	0.2
Hapmap31609-BTC-041148			38223329	R	A = 0.49	0.015	0.0170	0.4
HapMap23507-BTC-041133	✓	rs110961068	38233088	M	C = 0.45	0.00019	0.0192	1
Hapmap27082-BTC-041131			38233962	R	A = 0.44	-0.027	0.0178	0.1
Hapmap26855-BTC-041123			38239191	R	G = 0.42	0.025	0.0185	0.2
Hapmap23341-BTC-041113			38250098	K	T = 0.44	0.013	0.0181	0.5
81441_243			38255270	S	G = 0.46	0.017	0.0186	0.4
HapMap31285- BTC-041097	✓	rs109294917	38256889	Y	C = 0.44	0.021	0.0175	0.2
81435_188			38284737	Y	T = 0.42	-0.030	0.0189	0.1
**Hapmap28589-BTC-041045^6^**			**38300298**	**S**	**G = 0.45**	**0.038**	**0.0185**	**0.04**
Hapmap33629-BTC-041038			38313142	Y	T = 0.44	0.026	0.0191	0.2
Hapmap33694-BTC-041005			38342183	R	G = 0.08	0.021	0.0276	0.4
**81405_282^6^**			**38376731**	**M**	**A = 0.46**	**0.039**	**0.0180**	**0.03**
HapMap27529-BTC-050639	✓	rs110335851	38420476	R	G = 0.29	-0.013	0.0187	0.5
HapMap28546- BTC-072715	✓	rs43027303	38558526	S	G = 0.21	-0.034	0.0204	0.1
HapMap27537- BTC-060891	✓	rs110240246	38638962	R	A = 0.40	-0.0064	0.0218	0.8

^1 ^Name of publicly available markers from the following references: Van Tassell CP et al. [26], Bovine HapMap Consortium et al. [27], and Bovine Genome Sequencing and Analysis Consortium et al. [28].

^2 ^Markers with check marks are from the BovineSNP50 Illumina BeadChip. These markers were significantly associated with ADFI and/or ADG in the discovery population of crossbred steers [9].

^3 ^Position based on the Btau 4.0 Bovine Genome Assembly.

^4^NP corresponding to the effects presented.

^5 ^ADFI and ADG effects are displayed as kg/d. Effects are shown that correspond to the minor allele.

^6 ^Markers in bold font, Hapmap28589-BTC-041045 and 81405_282, were significantly associated with ADG in the validation population of animals (*P *= 0.04 and 0.03, respectively).

The two markers significantly associated with ADG in the validation population, Hapmap28589-BTC-041045 and 81405_282, are each responsible for 1.6% of the additive phenotypic variation of ADG (data not shown); however, they are in complete LD (Figure 1). These same markers each account for 3.6% of the phenotypic variation of ADG in the discovery population. These markers also represent 2% of the ADFI phenotypic variation in the discovery population of animals. These markers are also in complete LD in the discovery population of animals (Figure 2).

## Discussion

A previous single nucleotide polymorphism association analysis identified a 570 Kb region on BTA6 from 37.96 to 38.53 Mb containing eight SNP with significant association (*P *≤ 0.003) with ADG and three SNP with significant association with ADFI (*P *≤ 0.002) [9]. The three markers significant for ADFI were the same markers significant for ADG. The most significant marker in this region from Snelling et al. [9] for ADG was ARS-BFGL-NGS-45457 (*P*, nominal = 4.8 × 10^-6^) and the most significant marker for ADFI was Hapmap31285-BTC-041097 (*P*, nominal = 8 × 10^-4^). Of the markers presented in this study, 14 are more significant for ADFI (*P*, nominal = 5 × 10^-4^) and 11 were more significant for ADG (*P*, nominal = 4 × 10^-6^) than the markers on the BovineSNP50 BeadChip.

We chose to evaluate *LAP3, NCAPG *and *LCORL *as potential positional and functional candidate genes on bovine chromosome 6 for steer feed intake and gain. These genes were within the region that was identified as significant for ADFI and ADG from a previous association analysis [9]. None of the markers in *LAP3 *or in close proximity to *LAP3 *were significant after correction for multiple testing suggesting that these SNP are not in high LD with the causative mutation and that this region is not likely to be contributing to variation in feed intake and gain in this population of cattle. Our data do, however, suggest that the more likely region responsible for variation in cattle feed intake and gain is further downstream in the *NCAPG*-*LCORL *locus.

*NCAPG *is a subunit of the condensin 1 complex that is involved in the condensation of chromosomes and also interacts with a DNA methyltransferase linking methylation and chromatin condensation; however, the molecular and cellular events that might specifically tie *NCAPG *to growth and appetite have not been identified.

*LCORL *encodes a transcription factor that is thought to function during spermatogenesis. Aside from the association of SNPs in *LCORL *with human height phenotypes, there is little published information regarding this gene and its potential functions relating to growth. While it is possible to speculate that as a transcription factor, SNPs that affect the transcription or translation of *LCORL *may result in increased or decreased regulation of gene(s) involved in growth and/or appetite pathways, these functions have yet to be elucidated.

The *NCAPG-LCORL *locus has been interrogated in other populations of cattle for growth, meat quality and carcass traits. The most significant marker tested within the *NCAPG-LCORL *region in Japanese Black and Japanese Brown cattle for carcass weight, longissimus muscle area and subcutaneous fat thickness was the I422M marker (or marker 83714_224 in this study) in *NCAPG *[8]. This same marker was significant for fetal growth in a crossbred population of Charolais and German Holstein [11]. In our crossbred steer population consisting of seven continental breeds of beef cattle, this marker was significant for similar traits including, HCW, REA, AFT, and also for ADG and ADFI; however, markers located further downstream and within the *LCORL *gene locus were even more significant. The only markers tested in all three of these studies were in the *NCAPG *gene. Only one marker within the *LCORL *gene locus (HapMap28154-BTC-041134) was tested in our population and in the study by Eberlein et al. [11], making a direct comparison of the *LCORL *region difficult. In our population of cattle, we detected 14 SNP in the *NCAPG-LCORL *locus that were significant for ADFI, ADG and the carcass/meat quality traits HCW, AFT and REA. All of these SNP are in LD (r^2 ^≤ 0.73), thus it is unclear which of these SNP and genes may be responsible for the phenotypic variation within this population of animals.

In an attempt to better define which SNPs are responsible for the phenotypic variation, we performed a validation of these markers in another crossbred population of animals consisting of 14 breeds of cattle. This produced two SNP associated with ADG located in the intronic regions of the *LCORL *gene and suggests that these SNP may be robust among differing breeds of cattle for the prediction of weight gain. The LD in the validation population of animals in this region differs from the LD in the discovery population of animals. An example is that the LD for the *NCAPG *SNP 83714_224 (I422M) marker and the most significant SNP for ADG in our study (HapMap28589-BTC041045) was r^2 ^= 0.96 in our SNP discovery population and r^2 ^= 0.91 in the validation population.

The SNPs tested in our study and in previous work by Setoguchi et al. [8] are significant for traits that suggest they are involved in lean growth and fat deposition. In support of this, Gutiérrez-Gil et al. [2] also detected a QTL for subcutaneous fat in this region at BTA6:38.5, and McClure et al. [6] found a QTL for fat thickness at the 12^th ^rib at BTA6:36. These QTL plus the numerous QTL for growth and carcass weight seem to be harboring a gene or genes that are responsible for lean growth and fat deposition. Moreover, the *NCAPG *I442M SNP was associated with plasma arginine, symmetric dimethylarginine (SDMA), and linoleylcarnitine (C18:2) levels in a recent metabolomic study [29]. The allele that was associated with positive weight gain during 182-273 d (442M) was also associated with higher plasma levels of arginine, SDMA, and linoleylcarnitine [29]. The role of arginine in growth is through its activation of the mTOR pathway in skeletal muscle which increases protein synthesis [29,30], and through its function as a precursor of nitrogen oxide which effects glucose uptake and insulin responsiveness in skeletal muscle [29,31,32]. These data suggest there is some type of physiological role for growth via arginine for the gene or genes located within this chromosomal region. As the gene functions for *NCAPG *and *LCORL *do not currently seem to suggest a direct interaction with the metabolites identified in Weikard et al. [29], and the SNP in these genes are in high LD with each other in our discovery and validation populations of animals, additional functional genomic studies will be required to elucidate the mechanisms of *NCAPG *and/or *LCORL *in growth.

## Conclusion

In summary, we have identified several markers that are highly significant for steer feed intake and gain phentoypes. Moreover, several of the markers evaluated in this study are more significant for ADFI and ADG than the markers on the BovineSNP50 BeadChip. Genetic selection with these markers is likely to have an impact on HCW, REA and AFT with little influence on marbling or tenderness. It is not known how these markers will affect reproductive traits. These markers need to be evaluated on additional populations of animals to ensure that they maintain utility across breeds. We were able to confirm that two of these markers were significant for ADG in a validation population of crossbred cattle that included 14 breeds. These two markers may be particularly effective for the prediction of weight gain across various populations and breeds of cattle.

## Authors' contributions

ALP conceived of the study, carried out molecular genetic studies, and drafted the manuscript. AKS participated in the project design and carried out molecular genetic studies. LAK performed statistical analysis of genotypic data. WMS provided statistical advice. TPLS assisted with SNP selection. DAK, SDS and TLW managed meat quality and carcass phenotypic data collection. CLF, TGJ, HCF and LAK collected and provided feed efficiency phenotypic data. All authors read and approved the manuscript.
